# 173. Evaluating Respiratory Tract Culturing in Mechanically Ventilated Patients: Opportunity for Diagnostic Stewardship

**DOI:** 10.1093/ofid/ofac492.251

**Published:** 2022-12-15

**Authors:** Ravi Tripathi, Blaine Kenaa, Kimberly C Claeys, Meghana Patel, Mary Maldarelli, Michelle Newman, Surbhi Leekha

**Affiliations:** University of Maryland School of Medicine, Baltimore, Maryland; University of Maryland, school of Medicine, Baltimore, Maryland; University of Maryland Baltimore, Baltimore, MD; University of Maryland School of Medicine, Baltimore, Maryland; University of Maryland School of Medicine, Baltimore, Maryland; University of Maryland Baltimore, Baltimore, MD; University of Maryland School of Medicine, Baltimore, Maryland

## Abstract

**Background:**

Overdiagnosis of ventilator associated pneumonia (VAP) is common and may be due, in part, to excessive culturing of the respiratory tract in ventilated patients. We sought to evaluate the appropriateness of these cultures based on clinical characteristics leading to culture acquisition, and the relationship with antibiotic use.

**Methods:**

We included mechanically ventilated adult patients in the Neuroscience and Cardiac Surgery Intensive Care Units at an academic medical center in Baltimore, MD, who had respiratory tract cultures obtained from March 1 to June 30, 2021. The appropriateness of respiratory cultures was evaluated by chart review in a standardized manner using a published algorithm (Figure 1). Clinical characteristics of patients with appropriate vs. inappropriate cultures were compared using Fisher’s exact test.

**Figure:**
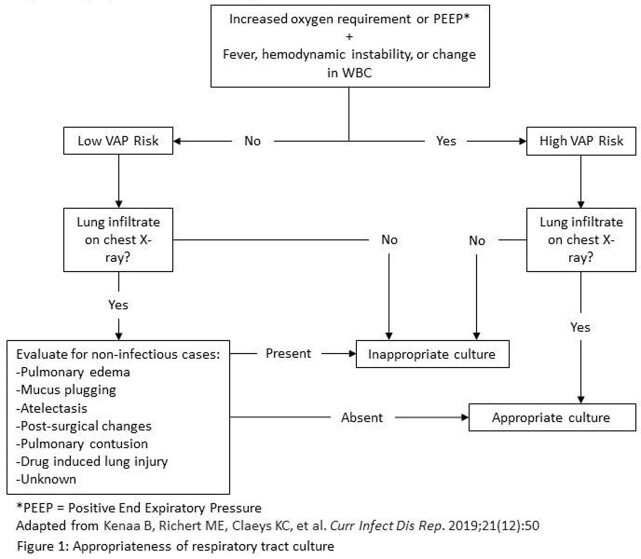

**Results:**

98 respiratory tract cultures (58 endotracheal aspirates, 25 mini bronchoalveolar lavages (BAL), 13 BALs, and 2 bronchial washings) were evaluated. Of these, 19 (19%) were deemed appropriate and 79 (81%) were inappropriate. Compared to patients with inappropriate cultures, those with appropriate cultures were more likely to demonstrate worsening oxygenation, new or increasing infiltrate on chest x-ray, or hemodynamic instability. The groups did not significantly differ with respect to fever or changes in WBC count (Figure 2). Of 79 inappropriate cultures, 31 (39%) received a clinical diagnosis of VAP from their provider. In 23 (29%) instances, antibiotics were started or changed based on culture results, and 9 (11%) were on empiric therapy for VAP that was continued unchanged. An additional 24 (30%) continued antibiotics not directed toward VAP. Excluding those on antibiotics not directed towards index respiratory culture results, patients received a median of 6 (IQR 2.5, 7) days of therapy.

**Figure:**
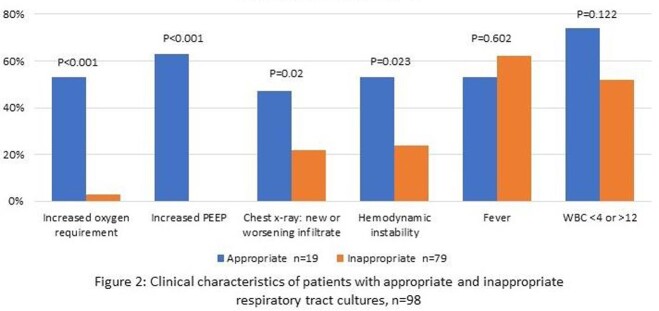

**Conclusion:**

A large majority of lower respiratory tract cultures in mechanically ventilated patients are potentially inappropriate and associated with overdiagnosis of VAP and unnecessary antibiotic exposure. Incorporating respiratory tract specific attributes into clinical decision making rather than “pan-culturing” for fever or leukocytosis may be an opportunity for diagnostic stewardship in this setting.

**Disclosures:**

**Kimberly C. Claeys, PharmD**, BioFire Diagnostics: Honoraria.

